# Nitroxides Mitigate Neutrophil-Mediated Damage to the Myocardium after Experimental Myocardial Infarction in Rats

**DOI:** 10.3390/ijms21207650

**Published:** 2020-10-16

**Authors:** Mary El Kazzi, Han Shi, Sally Vuong, Xiaosuo Wang, Belal Chami, Yuyang Liu, Benjamin S. Rayner, Paul K. Witting

**Affiliations:** 1 Discipline of Pathology, Charles Perkins Centre, Faculty of Medicine and Health, The University of Sydney, Sydney 2006 NSW, Australia; mael2199@uni.sydney.edu.au (M.E.K.); hshi2945@uni.sydney.edu.au (H.S.); x.wang2@victorchang.edu.au (X.W.); belal.chami@sydney.edu.au (B.C.); annaliu456@gmail.com (Y.L.); 2 Heart Research Institute, Sydney Medical School, The University of Sydney, Sydney 2006 NSW, Australia; svuo2980@uni.sydney.edu.au

**Keywords:** heart attack, neutrophil, host damage, cardiac damage

## Abstract

Reperfusion therapy increases survival post-acute myocardial infarction (AMI) while also stimulating secondary oxidant production and immune cell infiltration. Neutrophils accumulate within infarcted myocardium within 24 h post-AMI and release myeloperoxidase (MPO) that catalyses hypochlorous acid (HOCl) production while increasing oxidative stress and inflammation, thereby enhancing ventricular remodelling. Nitroxides inhibit MPO-mediated HOCl production, potentially ameliorating neutrophil-mediated damage. *Aim*: Assess the cardioprotective ability of nitroxide 4-methoxyTEMPO (4MetT) within the setting of AMI. *Methods*: Male Wistar rats were separated into 3 groups: SHAM, AMI/R, and AMI/R + 4MetT (15 mg/kg at surgery via oral gavage) and subjected to left descending coronary artery ligation for 30 min to generate an AMI, followed by reperfusion. One cohort of rats were sacrificed at 24 h post-reperfusion and another 28 days post-surgery (with 4MetT (15 mg/kg) administration twice daily). *Results*: 3-chlorotyrosine, a HOCl-specific damage marker, decreased within the heart of animals in the AMI/R + 4-MetT group 24 h post-AMI, indicating the drug inhibited MPO activity; however, there was no evident difference in either infarct size or myocardial scar size between the groups. Concurrently, MPO, NfκB, TNFα, and the oxidation marker malondialdehyde increased within the hearts, with 4-MetT only demonstrating a trend in decreasing MPO and TNF levels. Notably, 4MetT provided a significant improvement in cardiac function 28 days post-AMI, as assessed by echocardiography, indicating potential for 4-MetT as a treatment option, although the precise mechanism of action of the compound remains unclear.

## 1. Introduction

Coronary artery disease (CAD) is the leading cause of mortality worldwide [1] and ensues from the narrowing of the blood vessel lumen due to the accumulation of atherosclerotic plaques. A complication of CAD results from the rupture of the developing plaques, leading to a culprit thrombosis that manifests as a myocardial infarction [2]. The introduction of reperfusion therapy has presented a clinical paradox; on the one hand, it greatly contributes to the increase in survival rates post-acute myocardial infarction (AMI), due to relief of the blockage in the affected coronary artery and resumption of blood flow to the infarcted myocardium [3]. On the other hand, it results in reperfusion injury, consisting of a series of insults to the myocardium due to the production of reactive oxygen species (ROS), mitochondrial dysfunction, and immune cell infiltration and activation [4].

Neutrophils are the predominant infiltrating cell 24–48 h post myocardial reperfusion [5]; these infiltrating cells remove necrotic and extracellular matrix debris and stimulate the infiltration of inflammatory and then non-inflammatory macrophages, which act to resolve the ongoing inflammation and initiate the process of healing [6]. Neutrophils also release various proteolytic enzymes that are essential for the formation of non-contractile collagen scars, an essential component of myocardial healing post-AMI. Upon infiltration, neutrophils degranulate and release the enzyme myeloperoxidase (MPO), an enzyme that catalyses the formation of hypohalous acids, including hypochlorous acid (HOCl), the predominant product formed under physiological conditions. HOCl is a contributor to reperfusion injury, as it induces many downstream modifications to cellular targets which have the potential to impair myocardial functioning and increases oxidative stress in the myocardium. For example, the potent oxidant HOCl impairs the bioactivity of nitric oxide (NO) by uncoupling electron flow through damaging eNOS [7]. Oxidising HOCl also modifies thiol residues and many redox sensitive amino acids (cysteine, tyrosine, and methionine). For example, HOCl induces the chlorination of tyrosine residues, producing the biomarker 3-chlorotyrosine, which is used for quantification of HOCl-induced protein damage [8,9].

Neutrophil HOCl also oxidises cysteine thiol residues in the active site of matrix metalloproteinases (MMPs) [10] and the N-terminal cysteine in their inhibitors (TIMPs) [11]; these enzymes are essential for extracellular matrix (ECM) remodelling. This results in the overactivation of MMPs and deactivation of TIMPs, causing excessive ECM breakdown which results in excessive fibrosis and tissue remodelling, thereby impairing cardiac contractility and contributing to the progression to heart failure. Similarly, HOCl oxidises thiol residues in the active site of the antioxidants glutathione (GSH) and peroxiredoxins [12], which impairs the major endogenous antioxidant defence mechanisms in the myocardium, thereby rendering it more susceptible to oxidative stress and damage. Furthermore, HOCl and other hypohalous acids can dysregulate multiple intercellular signalling pathways, including kinase signalling (p38 and Erk2) [13] and inactivation of protein tyrosine phosphatase (PTP) [14]. HOCl can also influence cell viability, as cultured cardiomyocytes or endothelial cells demonstrate increased cell apoptosis and necrosis in the presence of HOCl [15,16]. Therefore, the modification of amino acid residues, disruption of the phosphoproteome balance, depletion of the antioxidant pool, increase in inflammation and fibrosis, and dysregulation of endothelial function all impair cellular and mitochondrial function and result in cytotoxicity.

Clinical studies have reported the role of MPO as a prognostic factor for mortality post-AMI [17,18], secondary cardiovascular complications, atrial fibrillation [19] and heart failure [20]. As such, due to the contribution of neutrophils to post-AMI injury, targeting this cell type may represent a promising way to limit this form of injury. However, neutrophils also play beneficial roles in the healing of the myocardium post AMI and therefore selective targeting of MPO and its downstream oxidants; most importantly, HOCl provides a better approach to decreasing HOCl-mediated cytotoxicity after reperfusion without impacting scar formation.

Nitroxides are a class of reversible MPO inhibitors that has received wide interest in the field of reperfusion therapy in the kidney [21], brain [22], intestinal [23], and ovarian tissue [24]. These cell permeable small molecules show low toxicity and are currently being used in vivo as therapeutics for radiation induced macular degeneration and alopecia [25]. They act via various mechanisms such as superoxide dismutase (SOD) mimetics, free radical scavengers, and reversible MPO inhibitors with catalytic self-regeneration [26,27]. Importantly, nitroxides inhibit HOCl production by acting as substrates for MPO compound I [28]. Nitroxides have been trialled within in vitro cultures of cardiomyoblast H9c2 cells exposed to activated neutrophils, where they were shown to alleviate the HOCl-induced increase in oxidative stress, phosphoproteome dysregulation, and apoptosis [14]. Accordingly, the current study aims to assess whether the administration of the nitroxide 4-methoxyTEMPO (4-MetT) will provide cardioprotection through inhibiting the neutrophil-MPO activity that occurs following AMI.

## 2. Materials and Methods

### 2.1. Chemicals

Chemicals used in the H&E and PSR stains were freshly prepared and of the highest quality. All other chemicals were of the highest purity and sourced from Sigma Aldrich (Castle Hill, NSW, Australia and St Louis, MO, USA), unless otherwise stated.

### 2.2. AMI Acute and Chronic Model

Male Wistar rats weighing 150–200 g were purchased from the Animal Resources Centre (Perth, WA, Australia) and were fed a diet of standard chow and housed in accordance with the local animal ethics guidelines for the South West Area Health Services Animal Ethics Committee (Protocol approval #2017/032; date of approval: date of approval: 1 November 2017) with an acclimation period of 1 week prior to surgical intervention. All cohorts were provided standard chow and water ad libitum for the duration of the study. Rats were split into respective cohorts, receiving a bolus dose of drug (15 mg/kg 4MetT) or equivalent volume of vehicle (0.9% saline) control (Sham and AMI/R) through oral gavage immediately prior to surgical intervention (acute study) and twice daily at 8 h intervals for the duration of the chronic study. To ensure tolerance of the dosing regimen and repeated animal handling, rat weights were recorded daily.

Anaesthesia was induced in an induction chamber (Advanced Anaesthesia Specialists, Gladesville, NSW, Australia) using 5% (*v*/*v*) vaporised isoflurane. The animal was then positioned supine and endotracheal intubation was performed using a blunt-end polyurethane 16-guage intravenous catheter fitted to a graduated 1 mL syringe. Anaesthesia was then maintained with 2% (*v*/*v*) isoflurane and the animals were ventilated at a rate of 75 breaths per min with a tidal volume of 10 mL per kg body weight of 0.2 mL O_2_/min using a small animal ventilator (Harvard, Holliston, MA). Immediately prior to surgery, an intramuscular injection of lignocaine (10 mg/kg body weight) and an i.p. injection of buprenorphine (0.1 mg/kg) were administered.

The upper thorax was then shaved, cleaned using Betadine antiseptic, and the chest opened at the 5th intercostal space with an incision parallel to the ribs. The heart was then exposed by spreading the ribs using a self-retaining retractor (World Precision Instruments, Sarasota, FL, USA). The pericardium was opened using sharp forceps and the left anterior descending (LAD) coronary artery was visualised and ligated using a 6/0 silk suture, which was passed through the myocardium deep to the vessel and through a snare to create a transient ligation for 30 min. Confirmation of vessel ligation was obtained through visualisation of blanching of the left ventricle anterior wall below the stitch. After 30 min, the snare was released, the suture removed from the heart, and reperfusion confirmed by the returned blood flow to the ischemic myocardium. The chest cavity was closed with layered sutures, a subcutaneous injection of lignocaine applied at the wound site, and the animal was revived and placed in an isolated chamber for recovery. Regular pain relief (10% buprenorphine solution in jelly) was provided for up to 72 h, commencing immediately upon the animal awakening. For the surgical Sham Control, the operative technique was identical except that no ligation of the LAD coronary artery was performed. Following either 24 h or 28 days of recovery, rats were anaesthetised as above, echocardiography was performed, and the hearts were excised for biochemical and histological analysis.

### 2.3. Echocardiogram Analysis of Functional Parameters

A transthoracic echocardiogram in the parasternal short axis position at the mid-ventricle level was performed at 24 h or 4 weeks post I/R injury prior to the rats being euthanised for subsequent tissue harvesting. Echocardiograms were performed using a SonoSite Edge II Ultrasound System (Bothell, WA, USA) using a HSL25x/13-6 MHz transducer (Fujifilm Sonosite, Bothell, WA, USA), viewed in M-mode to measure the LV end-systolic diameter (LVESD) and LV end-diastolic diameter (LVEDD). The mean measurements of three independent recordings performed in triplicate for each rat were used to calculate the fractional shortening (FS) and ejection fraction (EF) as detailed below:

FS (%) = [(LVEDD ‒ LVESD)/(LVEDD)] × 100

EF (%) = [(LVEDD^3^ ‒ LVESD^3^)/(LVEDD^3^)] × 100.

### 2.4. Biochemical Assays

Excised hearts were divided, and half the infarct tissue was homogenised using a rotating piston and Teflon coated glass tube (Wheaton, Millville, NJ, USA), as described in detail previously [29,30]. Hearts were homogenised in 1 mL lysis buffer [50 mM phosphate buffered saline (pH 7.4), 1 mM EDTA, 10 μm 2,6-Di-tert-butyl-4-methylphenol, 1 tablet/50 mL protease inhibitor cocktail (Roche, Basel, Switzerland), 1 tablet/10 mL PhosStop tablets (Roche, Basel, Switzerland)]. Total protein content of each aliquot was determined using a BCA assay as per the manufacturer’s instructions using Bicinchoninic acid assay [Sigma-Aldrich, Sydney Australia] in test samples and serially diluted bovine serum albumin (BSA)/MilliQ water to construct a standard concentration curve.

### 2.5. Liquid Chromatography-Mass Spectrometry (LC-MS) of 3-Cl-Tyr/Tyr Ratio

Levels of 3-chlorotyrosine measured relative to total Tyrosine levels (3-Cl-Tyr/Tyr) were measured using liquid chromatography coupled with mass spectrometry as described previously [31,32]. Proteins were precipitated with 0.3% *w/v* deoxycholic acid and 50% *w/v* trichloroacetic acid (TCA) and centrifuged at (7500× *g*; 5 °C, 2 min) to remove the supernatant. Pellets were washed in 5% *w/v* TCA, centrifuged, washed again in 100% ice cold acetone, and centrifuged. Isolated pellets were treated with 4M methanesulfonic acid and 0.2% *w/v* labelled tyrosine (13C9, 15N, L-Tyr; Cambridge isotope laboratories, Inc., Tewksbury, MA, USA) as well as a labelled chloro-tyrsine (13C9, 15N, L-Cl-Tyr; Sigma Aldrich, Sydney, Australia) internal standard mixture.

Next, the samples were placed in hydrolysis vessels (Eldex Laboratories, Napa, CA, USA) degassed with a vacuum pump, then were flushed with argon and heated at 110 °C for 16 h. Solid phase extraction was performed in chromatography extraction cartridges (Supelclean, Envi-Chrom, 250 mg, St. Louis, MO, USA) with 0.1% *w/v* TFA and 80% methanol *v/v* as the eluent. The eluate was dried in a vacuum centrifuge (Eppendorf, NSW, Australia, 60 °C, 3 h) then reconstituted in 0.1% *v/v* formic acid dispersed in HPLC grade water. Quantitative LC/MS was performed using a liquid chromatography triple quadrupole mass spectrometer 8050 (Shimadzu Corporation, Kyoto, Japan). Analyte separation was conducted using Agilent Zorbax Eclipse XDB-C181 (4.6 × 50 mm, 1.8 μm) with a UPLC Zorbax Eclipse XDB-C18 (4.6 × 50 mm, 1.8 μm) guard column. Eluent form the column was directed to a tandem for electrospray ionization MS with a dry gas flow of 10 L/min. All analytes were detected using multiple reaction monitoring (MRM) with using argon as the collision gas. Data was generated and analysed using the LabSolutions quantitative analysis software (Version 5.91, Shimadzu Corp, Kyoto, Japan).

### 2.6. Protein Tyrosine Phosphatase Activity Assay

Phosphatase activity was measured by a spectrophotometric assay as described in detail elsewhere [33]. This assay monitors the conversion of the colourless p-nitrophenyl phosphate (p-NPP) substrate to the yellow p-nitrophenyl (p-NP) product. Equal amounts of homogenate protein (5 μg from each sample as determined by BCA assay above) were diluted in 0.5 mM MgCl_2_ in PBS in a COSTAR 96 well flat bottom plate. p-NPP substrate (pH 7.4) was added to each well and absorbance readings were obtained at 405nm (every 20 min for 10 h, 37 °C) with a TECAN M200 PRO plate reader (Tecan, Austria). The concentration of p-NP produced was calculated using the Beer Lambert law: ΔA = εΔC_pNP_l with the path length (l) being 1 cm and the extinction coefficient for p-NP at 405 nm ε = 18 mM^−1^ cm^−1^. The rate of p-NP production in relation to time was plotted and phosphatase activity (nM/s) was determined by calculating the gradient of the plotted line during the first 80 min of the reaction.

### 2.7. Western Blot Analysis of p-p38

Levels of phosphorylated p38 were determined by Western blotting following protein separation by SDS-PAGE. Equal amounts of protein (5 μg) from each sample were diluted in MilliQ water, mixed with loading buffer (1/10 ß-mercaptoethanol in 4 × Laemmli sample buffer (Bio-Rad, Sydney, Australia), denatured at 95 °C for 5 min and loaded onto fast cast SDS-PAGE gels (TGX Stain-Free FastCast Acrylamide Kit; thickness 1 mm), which were prepared according to the manufacturer’s instructions. Proteins were resolved at 200 V for 45 min in resolving buffer [composition: 25 mM Tris, 192 mM glycine and 0.1% SDS]. Resolved proteins were imaged on a Bio-Rad ChemiDoc imaging system with Image LabTM software (version 5.2), which was used to calculate total protein content. Next, the resolved proteins were transferred onto an Immobilon Polyvinylidene Fluoride (PVDF; Millipore) membrane using a Bio-Rad Trans-Blot Turbo blotting system (Bio-Rad, Sydney, Australia) at (1 A; 25 V; 30 min) in transfer buffer [composition: 25 mM Tris, 190 mM glycine, 20% (*v*/*v*) methanol in 1 L of MilliQ water]. PVDF membranes were imaged on the Bio-Rad ChemiDoc imaging system.

Non-specific binding was minimised by incubating the membranes with 5% (*w*/*v*) BSA in Tris Buffered Saline with Tween-20 (TBST) [composition: 25 mM Tris base, 140 mM sodium chloride (NaCl), 4 mM potassium chloride (KCl), 0.1% Tween 20 in 1 L of MilliQ water] blocking solution on a rocker at 22 °C for 4 h. p-p38 was detected by incubation of the membrane with 1:1000 (*v*/*v*) MAPK phospho-p38 (p-p38; Cell Signalling technology) primary antibody in 1% (*w*/*v*) BSA in TBST. Excess primary antibody was washed, and binding antibody was labelled through incubation with 1:10,000 peroxidase labelled anti-rabbit IgG secondary antibody in 1% *w/v* BSA in TBST with rocking (22 °C; 1 h). Following washing steps, membranes were dipped in Immobilion Forte Western HRP substrate (Millipore) and proteins were visualised by chemiluminescence on the Bio-Rad ChemiDoc imaging system. Western Blot quantification was performed by using quantitative densitometry using Image Lab software by normalising p-p38 levels against corresponding total protein levels measured in the same lane imaged directly from membranes prior to transfer to PVDF, as described above. A ratio of the volume of p38 to total protein volume was calculated for each sample and divided by the protein-normalised ratio detected in the surgical SHAM group to obtain the fold-change in p38 phosphorylation in treatment groups relative to the surgical SHAM group.

### 2.8. GSH/GSSG Assessment

Levels of reduced glutathione (GSH) and glutathione disulphate (GSSG) were measured spectrophotometrically as described previously [34] by reaction of homogenates with 5,5′-dithio-bis (2-nitrobenzoic acid) (DTNB, 1.5 mg/mL) at 412 nm. To determine GSH levels; serial dilutions of GSH and GSSG were prepared from stock solutions in potassium phosphate EDTA buffer (KPE) and used to create a standard curve. Cell homogenates were prepared in 0.1 M KPE (pH 7.5). Blank KPE, GSH, and GSSG standards and cell homogenates in KPE were loaded into a COSTAR 96 well flat bottom plate. A mixture of DTNB: glutathione reductase (GR) solution (1:1 ratio) was added to each well for 30 s followed by β-NADPH; absorbance was read at 412 nm every 30 s for 2 min.

To determine GSSG levels, cell homogenates were pre-treated with 10% *v/v* 2-vinylpydrine (Sigma Aldrich, USA; 1 h; room temperature) then 10% *v/v* triethanolamine (TEAM, Sigma Aldrich, USA; pH 6–7; 10 min); absorbance was measured at 412 nm every 30 s for 2 min.

### 2.9. Histological Staining and Quantification of Infarct Size

Heart blocks were fixed in formalin, embedded in paraffin and sectioned on a rotary paraffin microtome (5 μm sections) to be used in all histological analysis. Slides were stained with haematoxylin and eosin to allow visualisation of infarcts and associated neutrophil infiltration. Slides were scanned using Zeiss AxioScan.Z1 at 20× magnification to obtain an image of the compete heart section. The infarct areas were quantified using QuPath software (v0.1.2). The infarct was outlined, and the total area of the infarct section was digitally determined by the software in μm^2^. The area of the ventricles was subtracted from the total area of the stained heart section to generate a relative area value for the full myocardial section. A normalised ratio of the infarct area: relative area was compared between all 3 groups. Infarct area quantification was performed by using two independent double-blinded assessors.

### 2.10. PSR Staining and Collagen Scar Quantification

Tissue sections were deparaffinised and rehydrated through a series of graded alcohols, immersed in a Picro Sirius red solution (22 °C; 1 h) and de-differentiated by dipping 3 times in acidified water. The slides were dehydrated, coverslipped, imaged on the ZEISS Axio Scan.Z1 Slide Scanner at 20× magnification, and collagen scar area was quantified using QuPath Software as described above.

### 2.11. TUNEL Staining of Non-Viable/Apoptotic Cell Levels

Cell viability in tissue sections was detected using a TUNEL fluorometric kit (Promega, NSW, Australia) according to the protocol supplied by the manufacturer. The slides were deparaffinised in xylene then rehydrated by immersion through a series of graded alcohols, washed in 0.85% *v/v* NaCl then PBS, fixed in 4% *v/v* paraformaldehyde in PBS, washed in PBS, and permeabilised in 20 μg/mL proteinase K (22 °C; 10 min).

Next, the slides were washed in PBS then fixed in 4% *v/v* paraformaldehyde, washed in PBS, and incubated with equilibration buffer (22 °C; 10 min) then with rTdT incubation buffer [90% Equilibration Buffer, 10% Nucleotide Mix, 2% rTdT Enzyme (37 °C; 1 h)]. Reaction was stopped by immersion in 2xSSC, slides were washed in PBS, and nuclei were counterstained in 1:600 Spectral DAPI (PerkinElmer, USA) in TBST. Fluorescent slides were imaged on the ZEISS AXIO SCOPE upright fluorescent microscope (Zeiss, NSW, Australia) and the level of cardiomyocyte viability was quantified using MetaMorph^®^ image analysis software (version 7.6; Molecular Devices, San Jose, CA, USA). An intensity threshold was manually selected to include all fluorescent apoptotic cells. The intensity of fluorescence was measured as integrated OD and normalised against the section area.

### 2.12. OPAL Multiplexing of Inflammatory Markers

OPAL multiplex immunohistochemistry allows the simultaneous staining of 6 different antigens of interest using the OPAL kit supplied by the manufacturer (PerkinElmer, Billerica, MA, USA). Thin tissue sections were deparaffinised in xylene then rehydrated by immersion through a series of graded alcohols. Antigen retrieval was performed in a pH6 retrieval buffer using a pressure cooker (125 °C, 30 s). The slides were washed in water and TBST, endogenous peroxidase activity was blocked (3% *v/v* H_2_O_2_; 22 °C; 5 min) and slides were washed again in TBST. Non-specific protein binding was minimised by incubation with a DAKO casein blocking agent (Agilent Technology, USA, 22 °C; 30 min) then slides were incubated with anti-MDA primary antibody (Abcam, VIC, Australia; 1:1000 *v/v* in antibody diluent; 22 °C; 1 h). Unbound antibody was washed with TBST (3 × 3 min) and primary antibody was labelled with Envision secondary antibody (DAKO, USA; 22 °C; 30 min). Slides were washed in TBST and incubated with the TSA fluorophore; OPAL690 (1:50 *v/v* in TSA diluent; 22 °C; 10 min). All subsequent steps were performed in the dark.

Excess TSA was washed with TBST and slides were microwaved in a pH9 retrieval buffer positioned in a water bath. The apparatus was heated (1000 W microwave, 100% power, 2 min), followed by 20 min of heating at 20% power. This microwaving process removed preformed complexes while leaving the bound fluorophore intact and the tissue ready for addition of the next antibody. This process was repeated for the following primary antibody and OPAL-fluorophore combinations: (i) pH9 retrieval buffer for the anti-p-p65 primary antibody (Abcam, Australia; 1:400 *v/v* in antibody diluent; 22 °C; 1 h) with OPAL620 (1:50 *v/v* in TSA diluent; 22 °C; 10 min); (ii) pH9 retrieval buffer for the anti-MAPK p-p38 primary antibody (Cell signalling, QLD, Australia; 1:200 *v/v* in antibody diluent; 22 °C; 1 h) with OPAL 570 (1:50 *v/v* in TSA diluent; 22 °C; 10 min) (iii) pH6 retrieval buffer for the anti-TNF primary antibody (Abcam, Australia; 1:500 *v/v* in antibody diluent; 22 °C; 1 h) with OPAL540 (1:50 *v/v* in TSA diluent; 22 °C; 10 min) employed as the secondary agent and (iv) pH6 retrieval buffer for the anti-MPO primary antibody (Abcam, Australia; 1:200 *v/v* in antibody diluent; 22 °C; 1 h) with OPAL520 (1:50 *v/v* in TSA diluent; 22 °C; 10 min).

Following a final wash, slides were incubated with 0.5% *w/v* Sudan Black B in 70% *v/v* ethanol (22 °C; 5 min) to decrease autofluorescence. Finally, slides were washed, and nuclei were counterstained with spectral DAPI (1:1000 *v/v* in TBST; 22 °C; 10 min), mounted with an aqueous mounting medium (DAKO, USA) and coverslips were applied. OPAL imaging was performed on a PerkinElmer MANTRA microscope (PerkinElmer, MA, USA) with a personal library that was developed using singleplex trials with the fluorophores to be imaged. The recorded spectrum for each fluorophore: OPAL690, OPAL620, OPAL570, OPAL540, OPAL520, and DAPI, was saved to be used when staining test tissue. Furthermore, an autofluorescence spectrum from each fluorophore was saved in the library. Upon image capture, a scale bar was added using the Fiji ImageJ software (version 1.52a) and OPAL quantification was performed using the ImageJ software. The number of cells expressing each marker was quantified and divided by the area of the imaged section expressed in pixels. However, due to the inconsistent staining of the marker MAPK p-p38 between the various myocardial samples from different treatment groups, it was not quantified.

### 2.13. Statistical Analysis

Statistical analysis of all experiments was performed on GraphPad Prism^®^; version 7. Where required raw data was tested for normality (distribution) using the Shapiro–Wilk test and Kolmogorov–Smirnov tests embedded in GraphPad with the alpha-value set to 0.05; these analyses indicated that data from AMI groups were normally distributed. Subsequently, all values were compared using one-way ANOVA with a *post hoc* Tukey test to correct for multiple comparisons or for pairwise comparisons, a Mann–Whitney test was used. Statistical significance was determined as *p* < 0.05. All graphed results were displayed as mean and standard deviation (SD) unless specified otherwise.

## 3. Results

### 3.1. Clinical Parameters

**Figure 1:**
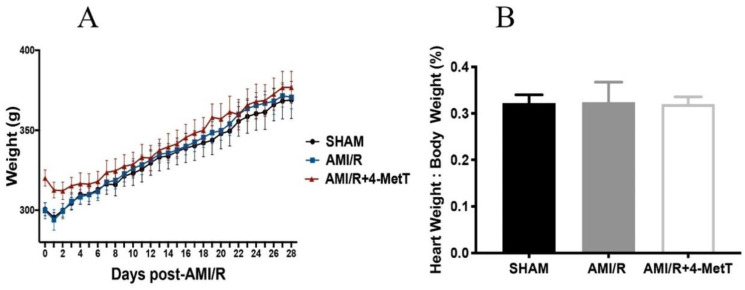
Change in the rat total weight and normalised heart weight 28 days after experimental acute myocardial infarction (AMI)-mediated IR-injury. Rats were separated into 3 groups: a surgical SHAM, rats subjected to experimental AMI, and rats supplemented with 4-Methoxy TEMPO immediately prior to experimental AMI (AMI/4-MetT group). (**A**) Rat weights were collected up to 28 days post-surgery. (**B**) Post harvesting of heart tissue from the rats, heart weight was collected and divided by total body weight of the animal at the time of collection. Data is presented as mean ± error bar (standard deviation; SD); SHAM *n* = 5, AMI/R *n* = 6, AMI/R+4-MetT drug treated *n* = 6.

In the chronic experimental model, rat body weight was measured daily to determine the time-dependent impact of the procedure and drug administration on the animals. No difference in weight was determined between the 3 treatment groups, signalling that both the procedure and the drug were well-tolerated. Immediately after organ harvest, heart weight was measured and normalised to the corresponding total body weight. No difference in the heart weight/body weight ratio was determined between the 3 groups, validating that both the procedure and the drug were well tolerated (Figure 1).

### 3.2. Echocardiography Analysis of Heart Function 4 Weeks Post-Experimental AMI

**Figure 2:**
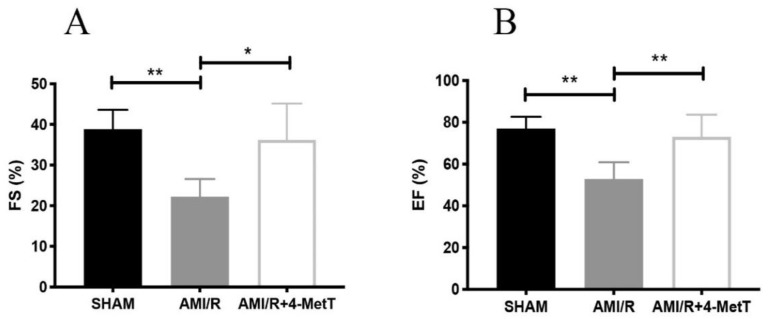
Fractional shortening and ejection fraction measured by echocardiography analysis is improved post 4-MetT administration in rats 4 weeks post experimental AMI-mediated IR-injury. Left ventricular function 4 weeks post-AMI/R was performed by echocardiogram analyses. The systolic and diastolic left ventricular diameters were measured by ultrasonography, as indicated in the Methods Section. Fractional shortening (**A**) and ejection fraction (**B**) were then calculated. Data is presented as mean ± error bar (standard deviation; SD); SHAM *n* = 5, AMI/R *n* = 4, AMI/R + 4-MetT drug treated *n* = 6. Different to the SHAM group; * *p* ≤ 0.05 or ** *p* ≤ 0.01. Abbreviations: FS; Fractional shortening, EF; Ejection fraction.

At 28 days post-experimental AMI, the contracting myocardium was imaged to measure the left ventricular diameter during systole and diastole. These measurements were used to calculate the ejection fraction (EF) and the fractional shortening (FS), established parameters of left ventricular function. Here FS (Figure 2A) and EF (Figure 2B) both decreased 4 weeks post experimental AMI/R while 4-MetT administration significantly restored these functional parameters to the baseline levels observed in the SHAM group. This outcome indicated that the nitroxide class of drugs can exhibit cardioprotection when provided at the time of experimental AMI with follow up daily administration. Next, we investigated whether this cardioprotection was linked to limiting neutrophil-MPO-mediated myocardial inflammation after experimental AMI.

### 3.3. Collagen Scar Size Quantification in the Different Treatment Groups

**Figure 3:**
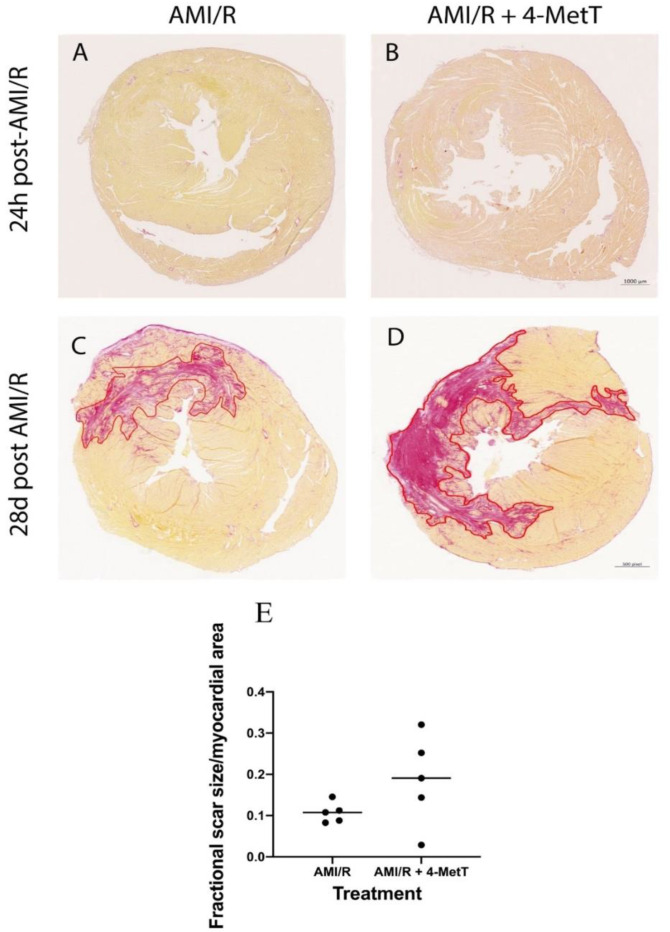
Scar size and the extent of fibrosis is not changed upon 4-MetT administration post-AMI/R. Thin paraffin embedded sections from the AMI/R and drug treated AMI/R + 4-MetT groups were stained with Picro Sirius Red as indicated in the Methods Section and sections from hearts isolated 24 h (**A**,**B**) and 28 days (**C**,**D**) post AMI/R were scanned to reveal collagen deposition (staining red) against yellow staining viable cardiomyocytes (scale bar = 1000 μm). SHAM panels were omitted, as they showed no variation in staining (not shown). (**E**) Slides were scanned with the ZEISS Axio Scan.Z1 Slide Scanner at 10× magnification and the collagen scar area was measured in μm^2^ using QuPath software and then normalised against the total myocardial area in the same stained section and compared between the two groups. Data was analysed using a Mann–Whitney (Rank test) and is presented as a distribution with the mean indicated as a horizontal line. (AMI/R *n* = 5, AMI/R + 4-MetT drug *n* = 5).

Cardiomyocytes are terminally differentiated cells, replaced after necrosis by a non-contractile collagen scar which stains red with Picro Sirius Red. As expected, tissues from rats sacrificed 24 h post AMI/R showed no collagen deposition, as it was too early for the fibrotic process to have initiated (Figure 3A,B). By contrast, 28 days post-surgery, a prominent red-staining collagen scar was visualised (Figure 3C,D). The collagen scar was quantified and normalised against the respective infarct size and showed a modest, non-significant increase in collagen deposition in the drug-treated animals compared to the AMI/R group in the absence of the nitroxide (Figure 3E), suggesting that the extent of fibrosis was similar in the two AMI groups independent of drug-treatment.

### 3.4. Quantification of 3-Cl-Tyr Levels

**Figure 4:**
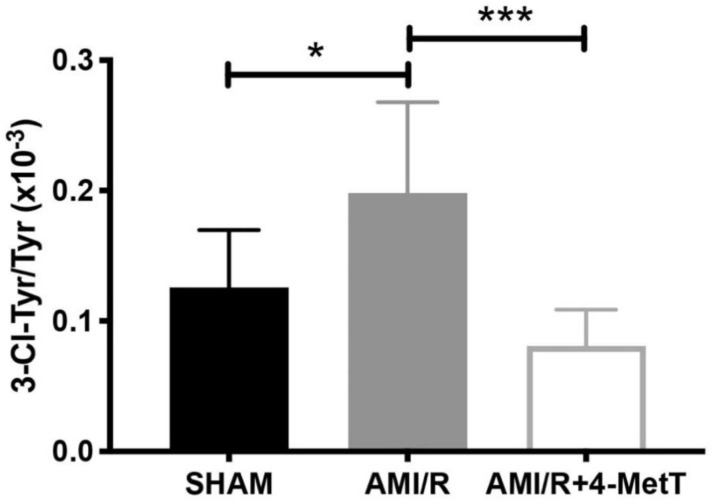
4-MetT significantly decreases HOCl-mediated production of 3-chlorotyrosine post experimental AMI/R. Myocardial levels of 3-chlorotyrosine and total tyrosine were assessed by using mass spectrometry coupled with liquid chromatography, as indicated in the Methods Section. Ratios of 3-chlorotyrosine/Tyrosine were compared between the 3 treatment groups. Data represent mean ± error bar (standard deviation; SD); SHAM *n* = 6; AMI/R *n* = 7; AMI/R + 4-MetT drug treated *n* = 8. * Different to the SHAM group *p* ≤ 0.05 or *** *p* ≤ 0.001.

Nitroxides interfere with the MPO enzymatic cycle and inhibit the production of HOCl. HOCl has a high affinity to tyrosine residues which it chlorinates generating the protein oxidation marker 3-Chlorotyrosine (3-Cl-Tyr); a widely employed specific in vivo biomarker for MPO-derived production of HOCl. As such, the capacity of 4-MetT to reach the target tissue and exert its protective functions was assessed by determining the levels of production of 3-Cl-Tyr as a marker of HOCl mediated damage to the heart. Analysis of the ratio of 3-Cl-Tyr/total tyrosine levels in homogenised heart tissues conducted by using liquid chromatography-mass spectrometry (representative mass spectra shown in Supplementary Figure S1) revealed a significant increase in 3-Cl-Tyr levels in the AMI group relative to the surgical sham (*p* < 0.05) (Figure 4). Notably, heart tissue from animals receiving 4-MetT showed significantly decreased 3-Cl-Tyr levels (~2-fold lower) relative to the corresponding untreated AMI group (*p* < 0.001) (Figure 4). Remarkably, administration of 4-MetT non-significantly decreased 3-Cl-Tyr levels to a level lower than the levels determined for the surgical SHAM group, indicating that the supplemented nitroxide, provided as a single gavage dose immediately prior to surgery, was efficient in inhibiting MPO activity in cardiac tissue.

### 3.5. Assessment of Thiol Oxidation

**Figure 5:**
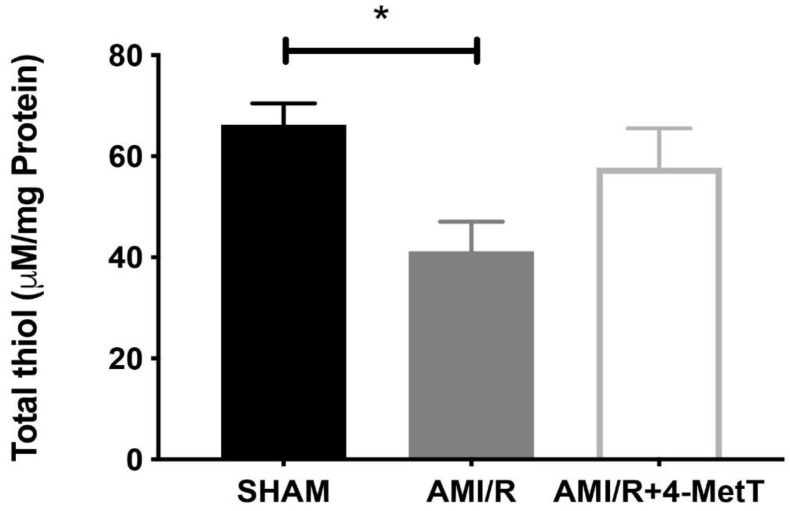
Total thiol levels in myocardial tissues. Total thiol levels representing low molecular weight reduced glutathione combined with thiol residues in proteins were measured by using a spectrophotometric assay which assessed the rate of hydrolysis of 2-nitrobenzoic acid (DTNB) into TNB, as indicated in the Methods Section. Data represent mean ± error bar (standard deviation; SD); SHAM *n* = 6; AMI/R *n* = 7; AMI/R + 4-MetT drug *n* = 8. * Different to the SHAM group *p* ≤ 0.05.

Following experimental AMI/R, total thiol levels which represent low molecular weight reduced glutathione (GSH) and protein thiol residues were significantly reduced compared to that of the SHAM group, whereas that of the AMI/R + 4-MetT group saw an increase in total thiol, although this did not reach statistical significance (Figure 5).

### 3.6. Alterations to Myocardial Kinase Activity after Experimental AMI

**Figure 6:**
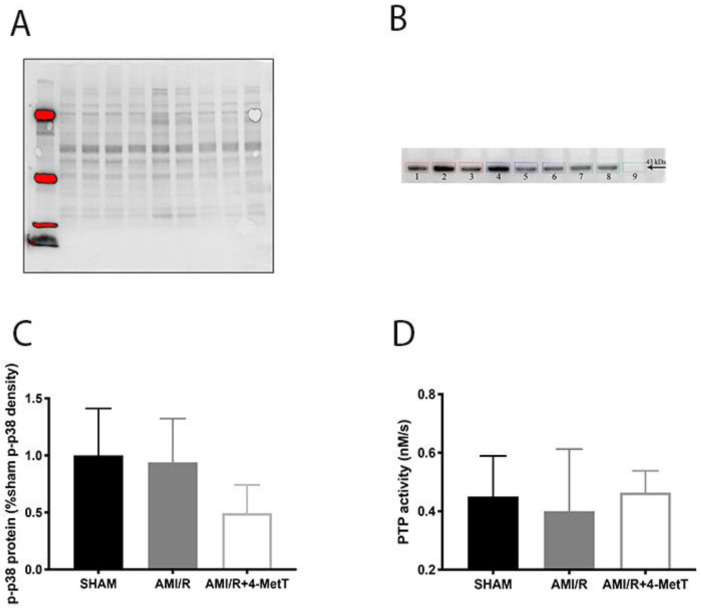
Phosphorylative activation of p38 post experimental AMI/R and 4-MetT administration**:** Levels of p-p38 were estimated by Western Blotting (total protein density blot and representative western blot shown in panels (**A**) and (**B**), respectively) and (**C**) quantified by normalising against total level of proteins by semi-quantitative densitometry and compared between the SHAM, AMI/R and AMI/R + 4-MetT treatment groups. (**D**) Protein tyrosine phosphatase activity was measured spectrophotometrically by assessing the extent of de-phosphorylation of the colourless p-nitrophenyl phosphate substrate that yields the yellow p-nitrophenyl product as monitored at 405 nm. (**D**) Total PTP activity was determined by calculating the rate of p-nitrophenyl production and comparing between the surgical SHAM, AMI/R and drug treated AMI/R + 4-MetT groups. Note the *x*-axis scale in panel B begins at 0.2 nM/s. Data represent mean ± error bar (standard deviation; SD). SHAM *n* = 6, AMI/R *n* = 6, AMI/R + 4-MetT drug *n* = 7.

Previous studies with cultured cardiomyocytes have reported that HOCl dysregulates the phosphoproteome by inhibiting cellular phosphatases and activating kinases, while nitroxides such as 4-MetT can restore this imbalance [14]. Hence, levels of myocardial p-p38 were investigated by using Western blotting to ascertain whether supplemented 4-MetT inhibited p38 activation in vivo. The loading control and corresponding protein blot are shown in Figure 6A,B, respectively, and protein density was quantified by quantitative densitometry. Overall, protein-normalised p-p38 levels in the AMI group were slightly lower than in the surgical SHAM group (94% vs. SHAM; *p* > 0.05; Figure 6C), although this did not reach statistical significance. Furthermore, a non-significant decrease in p-p38 levels was determined in hearts from the AMI/R + 4-MetT group in comparison to both the SHAM and AMI groups. Thus, p-p38 protein was ~1.9-fold lower in the AMI + 4-MetT group compared to the AMI group and represented 49% of the mean density in the SHAM group (Figure 6C).

To ascertain whether the supplemented nitroxide affected metabolism of phosphorylated proteins, total PTP activity was measured in the cardiac tissues. Note that PTPs contain a highly conserved thiol group in the active site that is highly susceptible to oxidative inactivation by HOCl. Thus, decreased total thiol levels in the tissue resulting from non-specific HOCl-oxidation may impact on PTP activity. However, only a small non-significant decrease in PTP activity is needed for the AMI group in comparison to SHAM to achieve a restoration of activity of PTP back to baseline post 4-MetT administration (Figure 6D).

### 3.7. Infarct Size Quantification in the Different Treatment Groups

**Figure 7:**
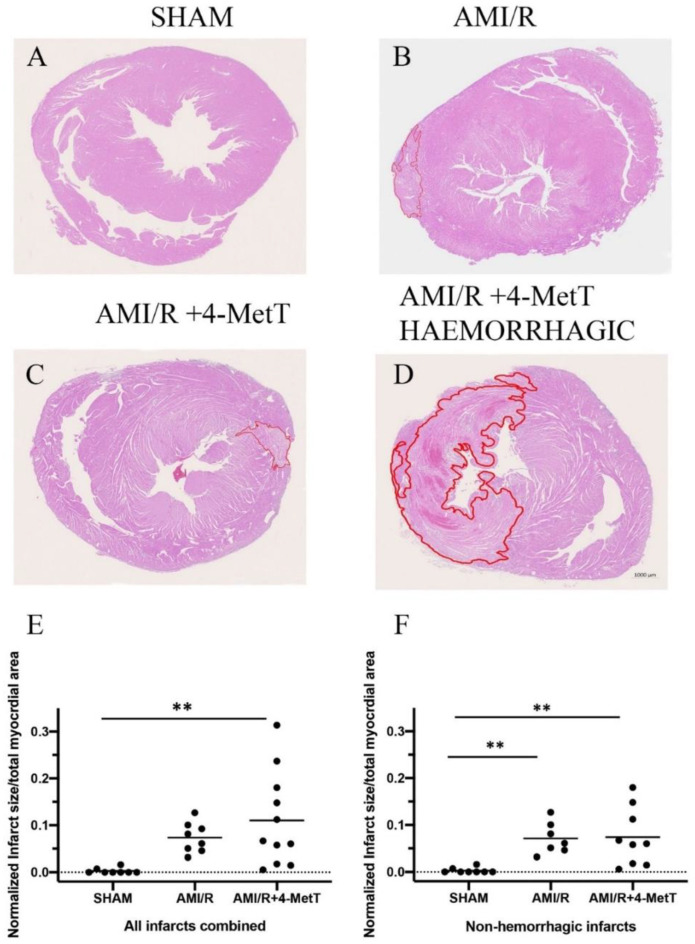
Infarct size visualised by haematoxylin and eosin staining is not changed upon 4-MetT administration post-AMI/R. Panels (**A**–**D**)—Thin paraffin embedded heart sections from the SHAM, AMI/R and drug treated AMI/R + 4-MetT groups were stained with Haematoxylin and eosin (SHAM *n* = 9, AMI/R *n* = 8, AMI/R + 4-MetT drug treated *n* = 11). Stained slides were imaged on the ZEISS Axio Scan.Z1 Slide Scanner to obtain an image of the entire heart section (scale bar = 1000 μm). The infarct region (circled in red) was defined by excessive apoptosis, cell damage, and neutrophil infiltration and measured in μm^2^ using QuPath software, then normalised against the total myocardial area in the same stained thin section. Calculations of infarct size ratios were then compared between all myocardial specimens in the 3 test groups (Panel (**E**); SHAM *n* = 9, AMI/R *n* = 8, AMI/R + 4-MetT drug *n* = 11), then compared again with the exclusion of infarcts that showed severe intramyocardial haemorrhage representative of extreme AMI phenotypes (Panel (**F**); SHAM *n* = 9, AMI/R *n* = 7, AMI/R + 4-MetT drug *n* = 9). Data is presented as a distribution with the mean indicated as a horizontal line. ** Different to the SHAM group; *p* ≤ 0.01.

As expected per the study design, no meaningful infarcts were detected in the SHAM group (Figure 7A), however these samples showed neutrophil infiltration into the pericardium due to the surgical process to expose the heart. The AMI/R (Figure 7B) and AMI/R + 4-MetT (Figure 7C) groups showed left ventricular infarcts of variable sizes characterised by excessive myocyte apoptosis, neutrophil infiltration, and diminished cytoplasmic staining due to the inability of the apoptotic cardiomyocytes to retain the eosin dye. Notably, some specimens from the AMI/R and the AMI/R + 4-MetT groups showed extensive intramyocardial haemorrhage (IMH) into the infarct region; characteristically, infarcts with IMH were larger than infarcts in the absence of haemorrhage across the AMI groups (Figure 7D). As such, infarct size/total myocardial area ratio was analysed in two different ways; (i) by the inclusion of all specimens containing a validated infarct (Figure 7E) and (ii) by the exclusion of infarcts displaying IMH (Figure 7F). This dual approach was taken to minimise any potential skewing of the data by infarcts displaying IMH. In the analysis of the complete cohort, the infarct size ratio increased in the AMI/R group as per the study design, while the AMI/R + 4-MetT group showed a marginally higher infarct size ratio, although this change was not significant relative to the AMI/R group in the absence the drug.

Re-analysis of the infarct size ratio with the exclusion of infarcts displaying IMH showed similar results with near identical changes in normalised infarct size for the AMI/R and AMI/R + 4-MetT groups relative to the SHAM group (*p* < 0.01 for both comparisons); the absolute level of this ratio however was smaller in this truncated group compared to the assessment in the complete cohort. However, irrespective of the method of analysis, 4-MetT failed to decrease the infarct size, suggesting that the drug may act through inhibiting processes subsequent to the primary damage.

### 3.8. Analysis of Myocardial Viability after Experimental AMI

**Figure 8:**
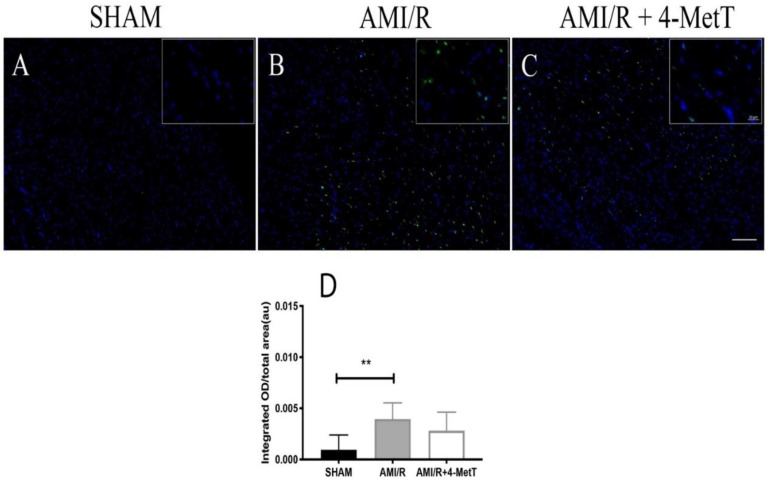
TUNEL labelling of apoptotic cells in heart sections of rat models of induced myocardial infarction and reperfusion. Thin paraffin embedded sections from the SHAM, AMI/R, and drug treated AMI/R + 4-MetT groups were labelled with a commercial kit; nuclei stained blue with DAPI (panel **A**) and TUNEL^+^ cells shown as a green fluorescence (panel **B**). Panel **C** shows the merged images. Tissue sections were permeabilised with Proteinase K, then incubated with the Terminal Deoxynucleotidyl Transferase, Recombinant, and an enzyme (rTdT) (supplied within the TUNEL kit, Promega) which catalyses the addition of fluorescein conjugated dUTP to fragmented DNA, and therefore TUNEL^+^ staining represents an assessment of cell viability. Slides were imaged at 10× (Panels **A**–**C**, scale bar = 200 μm) and 63× magnification (inset, scale bar = 20 μm) on a Zeiss AXIO SCOPE upright fluorescent microscope. Infarct size ratios were compared between all myocardial specimens in the 3 test groups with the exclusion of infarcts that showed severe intramyocardial haemorrhage representative of extreme AMI phenotypes (Panel (**D**); data shown is representative of 18 fields from SHAM (*n* = 9 samples), 42 fields from AMI/R (*n* = 7 samples) and 46 fields from AMI/R + 4-MetT (*n* = 7. samples**)**. Data represent mean ± error bar (standard deviation; SD) with *n*-values previously indicated. ** Different to the SHAM group; *p* ≤ 0.01.

The level of TUNEL^+^-staining (a surrogate for apoptosis) was analyzed and expressed as an integrated optical density and again normalised to areal fraction of the analyzed tissue. SHAM specimens showed minimal TUNEL^+^ staining (Figure 8A), while specimens in the AMI/R (Figure 8B) and AMI/R + 4-MetT (Figure 8C) both showed extensive TUNEL^+^ staining. In the truncated cohort, the number of non-viable TUNEL^+^ cells significantly increased in the AMI/R group in comparison to the SHAM group (*p* < 0.01). 4-MetT administration marginally decreased levels of non-viable cells; however, this did not reach statistical significance (Figure 8D).

### 3.9. Analysis of Inflammatory and Oxidative Biomarkers in the Infarcted Zone of Rat Hearts after Experimental AMI

**Figure 9:**
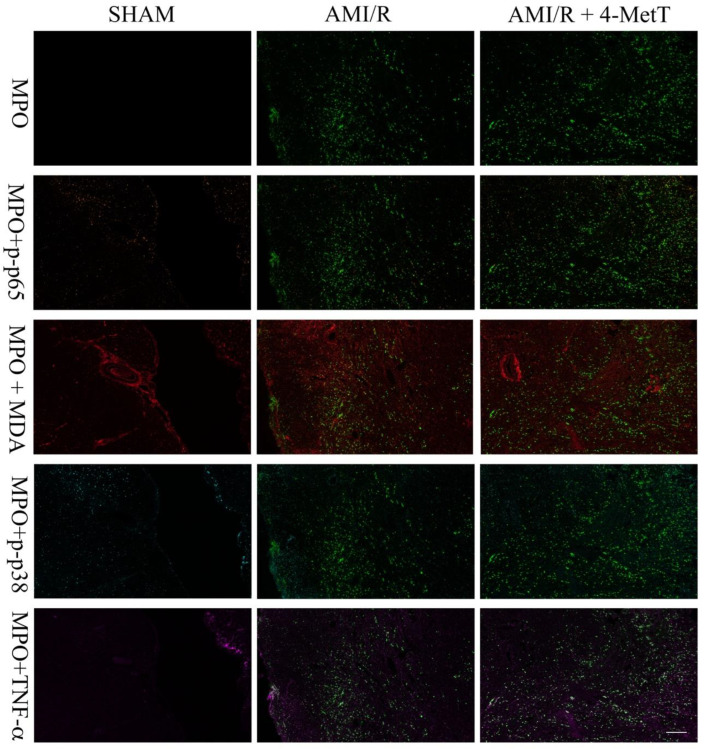
Immunofluorescence of inflammatory and oxidative markers in the infarct region from rats subjected to induced myocardial infarction and reperfusion. Multiplex immunohistochemistry was performed on thin (5 μm) paraffin embedded heart sections from a surgical SHAM, AMI/R, and AMI/R + 4-MetT groups. Slides were imaged on the MANTRA microscope at 10× power and prepared on the proprietary InForm software supplied with the MANTRA system. All fluorophores were imaged simultaneously, and desired markers were manually added or omitted. Myeloperoxidase staining was used as a marker for the infarct location (green). Nuclear factor kappa beta p65 subunit (orange), Malondialdehyde (MDA, red), mitogen activated protein kinase phospho-p38 (cyan), and Tumour necrosis factor alpha (magenta) are presented relative to the MPO defined infarct as markers for the extent of inflammation and oxidative stress within the infarct region. A scale bar (bottom right panel = 480 μm) was added using ImageJ software.

**Figure 10:**
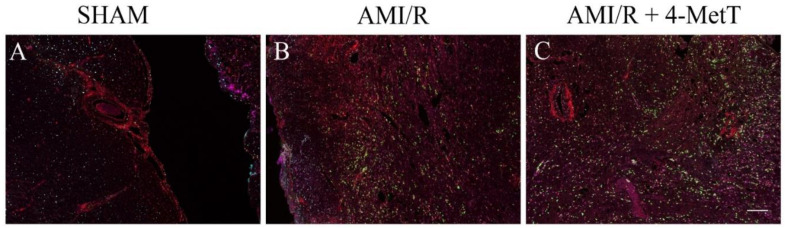
Multiplexed immunofluorescence of inflammatory and oxidative markers in the infarct region from rats subjected to induced myocardial infarction and reperfusion. Multiplex immunohistochemistry was performed on thin (5 μm) paraffin embedded heart sections from a surgical SHAM (panel **A**), AMI/R (panel **B**), and AMI/R + 4-MetT (panel **C**) groups. Tissue sections were stained for 5 different antigens malondialdehyde (red), nuclear factor kappa beta phosphorylated p65 subunit (orange), Mitogen activated protein kinase phosphorylated p38 (cyan), tumour necrosis factor alpha (magenta), and myeloperoxidase (green) with OPAL fluorophores. Successive heating (microwave, 1000 watts) of the slides between consecutive rounds of immunofluorescence removed previously formed complexes while leaving the attached fluorophores intact. Slides were imaged on the MANTRA PerkinElmer microscope and analysed on the InForm software. All fluorophores were imaged simultaneously and presented in this figure displaying the distribution of the stained oxidative and inflammatory antigens within the infarct and the degree of co-localisation. A scale bar (bottom right panel = 480 μm) was added using ImageJ software.

4-MetT treatment inhibited the MPO mediated production of HOCl in these heart tissues, therefore inflammatory (p-p38, p65, TNF) and oxidative markers (MDAs) were investigated simultaneously using multiplex immunohistochemistry (Figure 9). Leukocyte MPO was selected as the visual determinant of the infarct location (as neutrophils form a border around the primary infarct with simultaneous invasion into the infarct) and other markers were shown relative to MPO to visualise their localisation within the infarct. No meaningful MPO^+^-staining was detected in the surgical SHAM, whereas MPO^+^-immune fluorescence increased markedly in the AMI/R and nitroxide-treatment groups (Figure 9). MDA staining representative of oxidative damage-induced lipid peroxidation was minor in the surgical SHAM and markedly elevated in the remaining 2 groups (Figure 9). MDA^+^-immune fluorescence was elevated in areas with correspondingly high MPO^+^-immune fluorescence and relatively attenuated in areas of low MPO^+^-immune fluorescence suggesting a temporal relationship between MPO and oxidative damage in the heart tissues. Where imaged, vascular walls also displayed elevated MDA^+^ staining although this did not differ between the 3 groups. Inflammatory markers (MAPK p-p38, NfkB p65 and TNF) were present at low levels in the surgical SHAM and markedly elevated in the AMI/R and AMI/R + 4-MetT groups (Figure 9). Analysis of the multiplex image representing all 5 markers revealed co-localisation of MPO^+^ staining and staining of MDA and Nf-kB p65 (Figure 10). Interestingly, in the hemorrhagic infarcts, TNF-staining was most predominantly present at the border of the infarcts.

**Figure 11:**
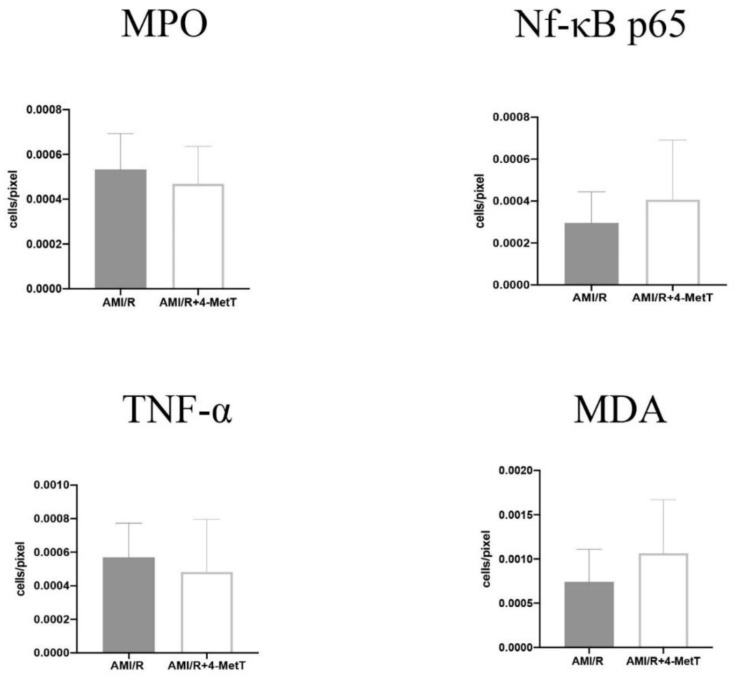
Quantitative analysis of the expression levels of inflammatory and oxidative markers stained with multiplex immunofluorescence in the infarct zone in non-haemorrhagic infarcts. Tissue sections stained by multiplex immunofluorescence were quantified using the ImageJ software. The number of cells expressing the designated marker were divided by the total area of imaged section in pixel. Multiple images of each section were quantified, and an average was obtained for each specimen. Infarcts showing intramyocardial haemorrhage and severe phenotype were excluded from this analysis. Data was analysed using an unpaired t-test and is presented as mean ± error bar (standard deviation; SD). Data shown is representative of 13 fields from SHAM (*n* = 7 samples), 25 fields from AMI/R (*n* = 7 samples), and 42 fields from AMI/R + 4-MetT (*n* = 8 samples).

Statistical analysis of the level of MPO^+^ and TNF^+^-immune fluorescent cells expressed per pixel of stained tissue revealed a non-significant trend to decreased levels of staining in the AMI/R + 4-MetT group compared to the AMI/R group, while levels of Nf-kB p65^+^ and MDA^+^ cells showed a non-significant increase in the expression of these markers in the drug treated group in comparison to the untreated AMI/R group (Figure 11). Therefore, no strong correlations between MPO and other inflammatory mediators were determined.

## 4. Discussions

One of the main contributors towards reperfusion injury is neutrophil infiltration that occurs within 24–48 h post AMI [5]. Upon activation, infiltrating neutrophils release MPO and this enzyme catalyses the formation of the oxidant HOCl that can elicit protein modifications, dysregulates the cellular phosphoproteome, deplete the GSH antioxidant pool, increases MMP activity, and subsequently promote fibrosis. As such, inhibiting MPO should alleviate HOCl mediated damage to the myocardium post-AMI. This study tested the efficacy of the MPO inhibitor and antioxidant 4-MetT in an experimental model of myocardial infarction. Echocardiogram analysis in rats revealed a significant restoration in EF and FS following daily administration of 4-MetT over 28 days. However, this functional restoration was evident irrespective of infarct size and degree of fibrosis seen in the collagen scar that remained the same in the presence or absence of the drug. Indeed, staining with Picro Sirius Red revealed that chronic administration of the drug failed to decrease the size of the collagen scar post-AMI.

By contrast, LC-MS analysis revealed that 3-Cl-tyrosine levels increased significantly 24 h post-experimental AMI, while 4-MetT administration significantly decreased this biomarker of MPO activity to levels below baseline (SHAM) levels. Given that 3-chlorotyrosine is an established marker of HOCl mediated damage, these results indicate that: (i) reperfusion post experimental AMI induces the activation of neutrophil-MPO to yield HOCl-mediated damage to cardiac tissue, and (ii) the supplemented nitroxide successfully reaches the myocardium and exerts its inhibitory activity on MPO, thereby decreasing MPO-activity (and HOCl production) and validating the study design. HOCl induces several protein modifications which alter the protein structure and result in altered protein function, which could result in depressed myocardial function. This is supported by previous studies which have shown that HOCl induces modifications of several proteins resulting in loss of function of these proteins. For example, HOCl oxidatively modifies horse heart myoglobin in vitro and results in loss of reduction of myoglobin from the ferric to the ferrous state caused by cytochrome b5 reductase, an event essential for oxygen transport caused by myoglobin [9]. Furthermore, HOCl and another MPO oxidant—HOSCN—modify low density lipoprotein (LDL), thereby interfering with endothelial vasorelaxation and inducing endothelial dysfunction [35]. Therefore, the drug might ameliorate these protein modifications, and this may account for the restoration of ventricular function observed 28 days after AMI was reported herein.

Infarct visualization by H&E revealed that specimens showed two different phenotypes: infarcts with a mild phenotype, and infarcts with a severe phenotype characterised by intramyocardial haemorrhage (IMH). Infarcts showing haemorrhage displayed excessive tissue damage and larger infarct sizes. This is consistent with studies reporting that IMH is associated with larger infarcts sizes, decreased myocardial salvage and, impaired left ventricular function in patients showing ST-elevation myocardial infarction (STEMI) and was associated with increased adverse cardiovascular events [36,37], however the small sample size in this study was insufficient to extrapolate any significant findings from this cohort and as such these specimens were excluded from all subsequent experiments. The pathogenesis of IMH remains unknown, however it has been suggested that the microvascular blockage of the vasculature during reperfusion injury decreases the integrity of the vascular walls, while reperfusion aggravates this permeability and enhances endothelial leakage into the infarcted tissue [38]. Nonetheless, 4-MetT failed to decrease the infarct size irrespective of the presence or absence of IMH. The effect of MPO inhibitors on modulating infarct size has been controversial as some MPO inhibitors decreased infarct size [39] while others failed to do so [40]. Given the association between infarct size and the degree of tissue apoptosis, myocardial viability was assessed by using TUNEL. Interestingly, the drug non-significantly restored myocardial viability and apoptosis levels tended to decrease in infarcts without IMH.

Other downstream markers dysregulated by HOCl were then tested and revealed that the reperfusion significantly depleted total thiols in the myocardium, while drug administration marginally restored levels close to baseline and in turn this correlated with slightly decreased p38 activation, although drug-mediated alterations in these parameters did not reach significance. Notably, MPO^+^-staining co-localised with MDA^+^ staining, thereby validating the role of neutrophils and HOCl in activating oxidative pathways in the myocardium post-AMI. However, the drug did not show any potential in decreasing the levels of oxidative damage and pro-inflammatory activation. A lack of efficacy for 4MetT in modulating myocardial inflammation and oxidative damage is somewhat contradictory to previous studies that have shown that the MPO inhibitor PF-1355 [40] and nitroxides [41] can decrease inflammation in models of ischemia and reperfusion. This discrepancy between the results could be attributed to the drug administration regimen, as the MPO inhibitor PF-1355 was administered for 7 and 21 days [40] while the nitroxide in the Zhu study discussed below was conjugated to a hydrogel ensuring longevity of the drug for a period of 1 week. In this study, the drug was administered once by oral gavage prior to surgery in the immunofluorescence analysed specimens.

While the nitroxide failed to reduce infarct size, the extent of myocardial fibrosis, and the inflammatory activation and oxidative damage, it significantly restored left ventricular function. This could be explained by the significant decrease in 3-chlorotyrosine levels post-drug administration. As explained above, tyrosine chlorination is a non-specific protein modification which could change the structure of a protein. If this modification is in a site essential for the activity of that protein, then such modification could result in increased or decreased activity. It is also important to consider that HOCl induces many other protein modifications upon release from activated neutrophils [42], and inhibition of these protein modifications and their disturbance of the protein function could account for the observed restoration of left ventricular function by 4-MetT. In support of this, Vasilyev et al. [43] reported that MPO induces the oxidation of amino acids such as glycine and threonine into cytotoxic aldehydes in vivo, which are involved in decreasing cardiac function and worsening of left ventricular remodelling. The authors further reported that genetic deletion of MPO in MPO knockout mice restored left ventricular function and attenuated LV remodelling. As such, this suggests that the functional restoration of ventricular function upon inhibition of MPO could be associated with a decrease in the oxidative modification of proteins essential for ventricular function as determined here.

Nitroxides are rapidly reduced to the hydroxylamine form [44] in vivo; however, in the presence of an oxidative environment such as post-AMI/R the hydroxylamine form can recycle back to the active nitroxide moiety. It is worth noting that in the acute model employed in this study, the drug was only delivered once by oral gavage, suggesting that a proportion could have been rapidly metabolised before being redistributed to the heart. This approach may have resulted in lower concentrations of active drug reaching the heart than established with the cellular model of cardio protection [14]. Therefore, future studies in this model could increase the oral drug dosage to achieve higher concentrations of active drug in the heart prior to assessing cardio-protection through the antioxidant and anti-inflammatory pathways we have assessed. This assertion is further supported by a novel study which has compared modes of delivery of a nitroxide either directly injected into the heart or delivered covalently bonded to a thermally responsive hydrogel which ensured drug longevity and prolonged drug scavenging properties [41]. The authors reported that the hydrogel conjugated nitroxide showed a decrease in IL-1b secretion, a decrease in apoptosis, and improved cardiac functioning measured as increased left ventricular end diastolic volume [41]. This suggests that a strategy to increase drug concentration and half-life results in an improved cardio-protection, and many studies are currently researching new ways to increase the potency of nitroxides in vivo through this mechanism [44,45].

### Study Limitations

Myocardial stunning (and preconditioning of myocardial tissue) may be an important issue that was not assessed here. Assessing myocardial stunning would require a functional heart assessment soon after reperfusion. However, in the current study design, ultrasound imaging to assess cardiac function was not performed until 24 h after reperfusion (at the earliest), corresponding to a time period where neutrophils are present in the cardiac tissue and the hypothesis that the drug 4-MetT acts to inhibit MPO can be reasonably tested.

Notably, elegant work by Roberto Bolli has suggested that free radical scavenging antioxidants can play a role in minimising myocardial stunning [46], so there is merit in testing whether 4-MetT diminishes myocardial stunning in addition to inhibiting MPO and corresponding HOCl-mediated tissue damage; the study could then test an additive protective effect (if any impact on stunning were evident). Therefore, an expanded study is warranted and should be considered in future experimental design. Alternative mechanisms to explain cardio-protection also include maintenance of the K(ATP) channel function [47], a role for nitric oxide [48], and protein tyrosine kinases [49] through a nuclear factor-kappaB pathway [50]. Thus, the current study is somewhat limited in scope and additional studies aiming to combine aspects of radical scavenging that impact on myocardial stunning, maintenance of K(ATP), assessment of NO levels, kinase activity, and transcriptional pathways that link with MPO-inhibition may well identify a complete and novel mechanism of action for this class of cyclic nitroxides.

Another limitation includes the lack of a scrambled (non-MPO targeting) treatment arm in the study design, although physiological saline was included as a non-drug (vehicle) control in the SHAM and AMI groups. Finally, although we reviewed PSR staining as a surrogate for the extent of myocardial scarring after AMI, it would be prudent to review whether 4-MetT acts directly on myofibroblasts that heavily populate the primary infarct zone and are responsible for the overproduction of extracellular matrix through a mechanism involving TGF-beta [51].

## 5. Conclusions

The data reported herein using heart tissue from animal model of AMI/R suggests that supplementation with 4-MetT, a nitroxide which inhibits HOCl-mediated tyrosine residue modifications, can restore ventricular function and this may have a beneficial impact on longer term complications associated with surviving AMI. This cardioprotective action occurs in parallel with a decrease in HOCl-mediated protein tyrosine post-translational modification but is independent of alterations to infarct size and the extent of cardiac fibrosis. Further studies are required to assess the effects of HOCl (and mitigation of HOCl production by 4-MetT) on other protein modifications and other relevant molecular pathways in order to determine whether the cyclic nitroxides indeed have a role in the restoration of ventricular function.

## Supplementary Materials

The following are available online at https://www.mdpi.com/1422-0067/21/20/7650/s1.

## References

[1] Pagidipati N.J., Gaziano T.A. (2013). Estimating deaths from cardiovascular disease: A review of global methodologies of mortality measurement. Circulation.

[2] Davies M.J., Thomas A.C. (1985). Plaque fissuring—The cause of acute myocardial infarction, sudden ischaemic death, and crescendo angina. Br. Heart J..

[3] Bhindi R., Witting P.K., McMahon A.C., Khachigian L.M., Lowe H.C. (2006). Rat models of myocardial infarction. Pathogenetic insights and clinical relevance. Thromb. Haemost..

[4] Neuzil J., Rayner B.S., Lowe H.C., Witting P.K. (2005). Oxidative stress in myocardial ischaemia reperfusion injury: A renewed focus on a long-standing area of heart research. Redox Rep..

[5] Yan X., Anzai A., Katsumata Y., Matsuhashi T., Ito K., Endo J., Yamamoto T., Takeshima A., Shinmura K., Shen W. (2013). Temporal dynamics of cardiac immune cell accumulation following acute myocardial infarction. J. Mol. Cell Cardiol..

[6] Prabhu S.D., Frangogiannis N.G. (2016). The Biological Basis for Cardiac Repair after Myocardial Infarction: From Inflammation to Fibrosis. Circ. Res..

[7] Stocker R., Huang A., Jeranian E., Hou J.Y., Wu T.T., Thomas S.R., Keaney J.F. (2004). Hypochlorous acid impairs endothelium-derived nitric oxide bioactivity through a superoxide-dependent mechanism. Arterioscler. Thromb. Vasc. Biol..

[8] Kettle A.J. (1996). Neutrophils convert tyrosyl residues in albumin to chlorotyrosine. FEBS Lett..

[9] Ahmad G., Chami B., El Kazzi M., Wang X., Moreira M.T.S., Hamilton N., Maw A.M., Hambly T.W., Witting P.K. (2019). Catalase-Like Antioxidant Activity is Unaltered in Hypochlorous Acid Oxidized Horse Heart Myoglobin. Antioxidants (Basel).

[10] Fu X., Kassim S.Y., Parks W.C., Heinecke J.W. (2001). Hypochlorous acid oxygenates the cysteine switch domain of pro-matrilysin (MMP-7). A mechanism for matrix metalloproteinase activation and atherosclerotic plaque rupture by myeloperoxidase. J. Biol. Chem..

[11] Wang Y., Rosen H., Madtes D.K., Shao B., Martin T.R., Heinecke J.W., Fu X. (2007). Myeloperoxidase inactivates TIMP-1 by oxidizing its N-terminal cysteine residue: An oxidative mechanism for regulating proteolysis during inflammation. J. Biol. Chem..

[12] Stacey M.M., Vissers M.C., Winterbourn C.C. (2012). Oxidation of 2-cys peroxiredoxins in human endothelial cells by hydrogen peroxide, hypochlorous acid, and chloramines. Antioxid. Redox Signal..

[13] Midwinter R.G., Vissers M.C., Winterbourn C.C. (2001). Hypochlorous acid stimulation of the mitogen-activated protein kinase pathway enhances cell survival. Arch. Biochem. Biophys..

[14] Chami B., Jeong G., Varda A., Maw A.M., Kim H.B., Fong G.M., Simone M., Rayner B.S., Wang X.S., Dennis J.M. (2017). The nitroxide 4-methoxy TEMPO inhibits neutrophil-stimulated kinase activation in H9c2 cardiomyocytes. Arch. Biochem. Biophys..

[15] Reyes L., Hawkins C.L., Rayner B.S. (2019). Characterization of the cellular effects of myeloperoxidase-derived oxidants on H9c2 cardiac myoblasts. Arch. Biochem. Biophys..

[16] Lloyd M.M., Grima M.A., Rayner B.S., Hadfield K.A., Davies M.J., Hawkins C.L. (2013). Comparative reactivity of the myeloperoxidase-derived oxidants hypochlorous acid and hypothiocyanous acid with human coronary artery endothelial cells. Free Radic. Biol. Med..

[17] Arruda-Olson A.M., Reeder G.S., Bell M.R., Weston S.A., Roger V.L. (2009). Neutrophilia predicts death and heart failure after myocardial infarction: A community-based study. Circ. Cardiovasc. Qual. Outcomes.

[18] Ghaffari S., Nadiri M., Pourafkari L., Sepehrvand N., Movasagpoor A., Rahmatvand N., Rezazadeh Saatloo M., Ahmadi M., Nader N.D. (2014). The predictive Value of Total Neutrophil Count and Neutrophil/Lymphocyte Ratio in Predicting In-hospital Mortality and Complications after STEMI. J. Cardiovasc. Thorac. Res..

[19] Rudolph V., Andrie R.P., Rudolph T.K., Friedrichs K., Klinke A., Hirsch-Hoffmann B., Schwoerer A.P., Lau D., Fu X., Klingel K. (2010). Myeloperoxidase acts as a profibrotic mediator of atrial fibrillation. Nat. Med..

[20] Kyne L., Hausdorff J.M., Knight E., Dukas L., Azhar G., Wei J.Y. (2000). Neutrophilia and congestive heart failure after acute myocardial infarction. Am. Heart J..

[21] Zhang G., Wang Q., Zhou Q., Wang R., Xu M., Wang H., Wang L., Wilcox C.S., Liu R., Lai E.Y. (2016). Protective Effect of Tempol on Acute Kidney Injury Through PI3K/Akt/Nrf2 Signaling Pathway. Kidney Blood Press. Res..

[22] Kato N., Yanaka K., Hyodo K., Homma K., Nagase S., Nose T. (2003). Stable nitroxide Tempol ameliorates brain injury by inhibiting lipid peroxidation in a rat model of transient focal cerebral ischemia. Brain Res..

[23] Teke Z., Kabay B., Ozden A., Yenisey C., Bir F., Demirkan N.C., Bicakci T., Erdem E. (2008). Effects of tempol, a membrane-permeable radical scavenger, on local and remote organ injuries caused by intestinal ischemia/reperfusion in rats. J. Surg. Res..

[24] Pinar N., Soylu Karapinar O., Ozcan O., Atik Dogan E., Bayraktar S. (2017). Protective effects of tempol in an experimental ovarian ischemia-reperfusion injury model in female Wistar albino rats. Can. J. Physiol. Pharmacol..

[25] Zarling J.A., Brunt V.E., Vallerga A.K., Li W., Tao A., Zarling D.A., Minson C.T. (2015). Nitroxide pharmaceutical development for age-related degeneration and disease. Front. Genet..

[26] Lewandowski M., Gwozdzinski K. (2017). Nitroxides as Antioxidants and Anticancer Drugs. Int. J. Mol. Sci..

[27] Offer T., Mohsen M., Samuni A. (1998). An SOD-mimicry mechanism underlies the role of nitroxides in protecting papain from oxidative inactivation. Free Radic. Biol. Med..

[28] Rees M.D., Bottle S.E., Fairfull-Smith K.E., Malle E., Whitelock J.M., Davies M.J. (2009). Inhibition of myeloperoxidase-mediated hypochlorous acid production by nitroxides. Biochem. J..

[29] Groebler L.K., Wang X.S., Kim H.B., Shanu A., Hossain F., McMahon A.C., Witting P.K. (2012). Cosupplementation with a synthetic, lipid-soluble polyphenol and vitamin C inhibits oxidative damage and improves vascular function yet does not inhibit acute renal injury in an animal model of rhabdomyolysis. Free Radic. Biol. Med..

[30] Shanu A., Groebler L., Kim H.B., Wood S., Weekley C.M., Aitken J.B., Harris H.H., Witting P.K. (2013). Selenium inhibits renal oxidation and inflammation but not acute kidney injury in an animal model of rhabdomyolysis. Antioxid. Redox Signal..

[31] Talib J., Pattison D.I., Harmer J.A., Celermajer D.S., Davies M.J. (2012). High plasma thiocyanate levels modulate protein damage induced by myeloperoxidase and perturb measurement of 3-chlorotyrosine. Free Radic. Biol. Med..

[32] Hawkins C.L., Morgan P.E., Davies M.J. (2009). Quantification of protein modification by oxidants. Free Radic. Biol. Med..

[33] Montalibet J., Skorey K.I., Kennedy B.P. (2005). Protein tyrosine phosphatase: Enzymatic assays. Methods.

[34] Rahman I., Kode A., Biswas S.K. (2006). Assay for quantitative determination of glutathione and glutathione disulfide levels using enzymatic recycling method. Nat. Protoc..

[35] Abdo A.I., Rayner B.S., van Reyk D.M., Hawkins C.L. (2017). Low-density lipoprotein modified by myeloperoxidase oxidants induces endothelial dysfunction. Redox Biol..

[36] Kandler D., Lucke C., Grothoff M., Andres C., Lehmkuhl L., Nitzsche S., Riese F., Mende M., de Waha S., Desch S. (2014). The relation between hypointense core, microvascular obstruction and intramyocardial haemorrhage in acute reperfused myocardial infarction assessed by cardiac magnetic resonance imaging. Eur. Radiol..

[37] Eitel I., Kubusch K., Strohm O., Desch S., Mikami Y., de Waha S., Gutberlet M., Schuler G., Friedrich M.G., Thiele H. (2011). Prognostic value and determinants of a hypointense infarct core in T2-weighted cardiac magnetic resonance in acute reperfused ST-elevation-myocardial infarction. Circ. Cardiovasc. Imaging.

[38] Hollander M.R., de Waard G.A., Konijnenberg L.S., Meijer-van Putten R.M., van den Brom C.E., Paauw N., de Vries H.E., van de Ven P.M., Aman J., Van Nieuw-Amerongen G.P. (2016). Dissecting the Effects of Ischemia and Reperfusion on the Coronary Microcirculation in a Rat Model of Acute Myocardial Infarction. PLoS ONE.

[39] McDonald M.C., Zacharowski K., Bowes J., Cuzzocrea S., Thiemermann C. (1999). Tempol reduces infarct size in rodent models of regional myocardial ischemia and reperfusion. Free Radic. Biol. Med..

[40] Ali M., Pulli B., Courties G., Tricot B., Sebas M., Iwamoto Y., Hilgendorf I., Schob S., Dong A., Zheng W. (2016). Myeloperoxidase Inhibition Improves Ventricular Function and Remodeling After Experimental Myocardial Infarction. JACC Basic Transl. Sci..

[41] Zhu Y., Matsumura Y., Velayutham M., Foley L.M., Hitchens T.K., Wagner W.R. (2018). Reactive oxygen species scavenging with a biodegradable, thermally responsive hydrogel compatible with soft tissue injection. Biomaterials.

[42] Davies M.J. (2011). Myeloperoxidase-derived oxidation: Mechanisms of biological damage and its prevention. J. Clin. Biochem. Nutr..

[43] Vasilyev N., Williams T., Brennan M.L., Unzek S., Zhou X., Heinecke J.W., Spitz D.R., Topol E.J., Hazen S.L., Penn M.S. (2005). Myeloperoxidase-generated oxidants modulate left ventricular remodeling but not infarct size after myocardial infarction. Circulation.

[44] Kajer T.B., Fairfull-Smith K.E., Yamasaki T., Yamada K., Fu S., Bottle S.E., Hawkins C.L., Davies M.J. (2014). Inhibition of myeloperoxidase- and neutrophil-mediated oxidant production by tetraethyl and tetramethyl nitroxides. Free Radic. Biol. Med..

[45] Thomas K., Moody T.W., Jensen R.T., Tong J., Rayner C.L., Barnett N.L., Fairfull-Smith K.E., Ridnour L.A., Wink D.A., Bottle S.E. (2018). Design, synthesis and biological evaluation of hybrid nitroxide-based non-steroidal anti-inflammatory drugs. Eur. J. Med. Chem..

[46] Bolli R. (1998). Causative role of oxyradicals in myocardial stunning: A proven hypothesis. A brief review of the evidence demonstrating a major role of reactive oxygen species in several forms of postischemic dysfunction. Basic Res. Cardiol..

[47] Takano H., Tang X.L., Bolli R. (2000). Differential role of K(ATP) channels in late preconditioning against myocardial stunning and infarction in rabbits. Am. J. Physiol. Heart Circ. Physiol..

[48] Shinmura K., Tang X.L., Takano H., Hill M., Bolli R. (1999). Nitric oxide donors attenuate myocardial stunning in conscious rabbits. Am. J. Physiol..

[49] Dawn B., Xuan Y.T., Qiu Y., Takano H., Tang X.L., Ping P., Banerjee S., Hill M., Bolli R. (1999). Bifunctional role of protein tyrosine kinases in late preconditioning against myocardial stunning in conscious rabbits. Circ. Res..

[50] Xuan Y.T., Tang X.L., Banerjee S., Takano H., Li R.C., Han H., Qiu Y., Li J.J., Bolli R. (1999). Nuclear factor-kappaB plays an essential role in the late phase of ischemic preconditioning in conscious rabbits. Circ. Res..

[51] Salvarani N., Maguy A., De Simone S.A., Miragoli M., Jousset F., Rohr S. (2017). TGF-beta1 (Transforming Growth Factor-beta1) Plays a Pivotal Role in Cardiac Myofibroblast Arrhythmogenicity. Circ. Arrhythm. Electrophysiol..

