# Supplementary material for: Nitroxides Mitigate Neutrophil-Mediated Damage to the Myocardium after Experimental Myocardial Infarction in Rats
Source: Int J Mol Sci. 2020 Oct 16;21(20):7650. doi: 10.3390/ijms21207650 (PMC7589606; doi:10.3390/ijms21207650)
Supplement: Supplementary file 1 [file ijms-21-07650-s001.pdf]

A Role of Nitroxide Drugs in Mitigating Neutrophil Mediated Damage to the Heart after Experimental Myocardial Infarction?

Mary El Kazzi[1], Han Shi[1], Sally Vuong[2], Xiasuo Wang,[1] Belal Chami[1], Yuyang Liu[1], Ben Rayner[2] and Paul K Witting[1]

[1]Discipline of Pathology, Charles Perkins Centre, Faculty of Medicine and Health, The University of Sydney, NSW, 2006 Australia;
[2]Heart Research Institute, Sydney Medical School, The University of Sydney, NSW, 2006 Australia;

Article type: original article contribution.

Corresponding authors: Professor Paul K Witting, Discipline of Pathology, Charles Perkins Centre, Sydney Medical School, The University of Sydney, NSW, 2006, Australia; Ph 61-2-9114-0524; e-mail: paul.witting@sydney.edu.au

Key words: heart attack, neutrophil, host damage, cardiac damage

Supplementary Data

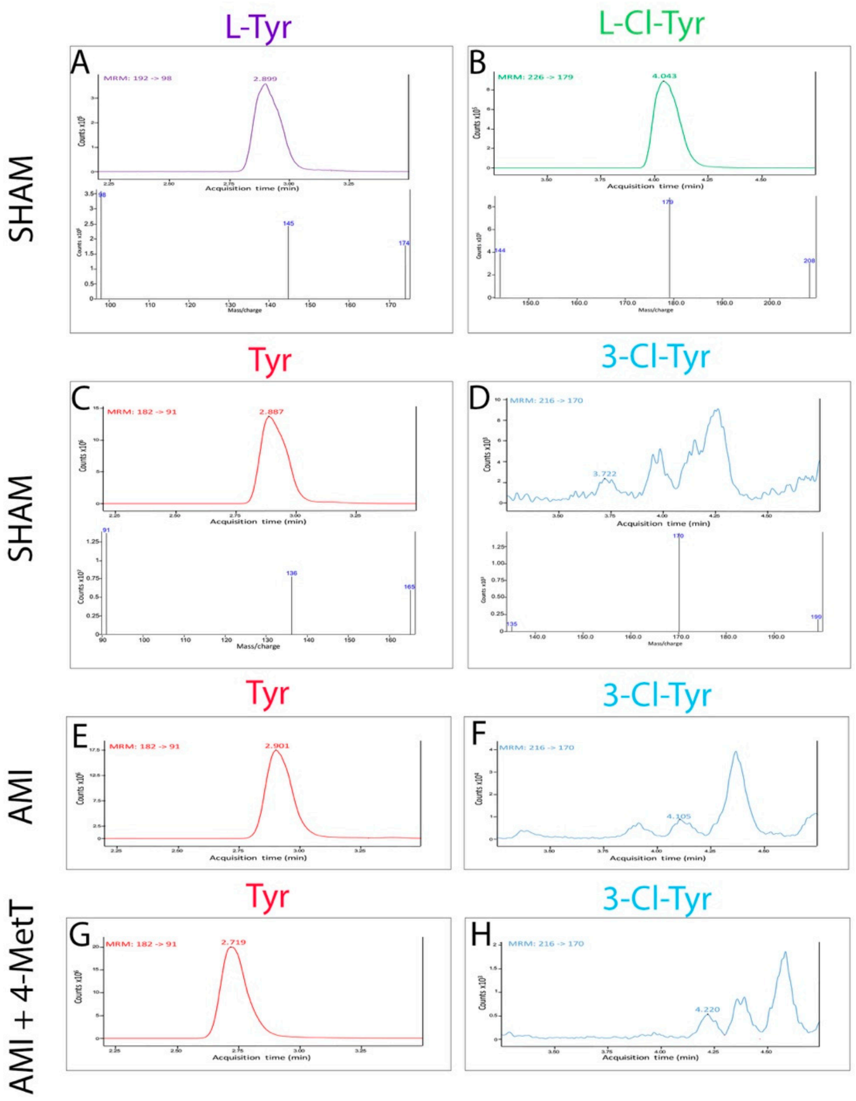

Supplementary Figure 1: Liquid chromatography-mass spectrometry analysis of 3-chlorotyrosine/Tyrosine ratio in rat hearts subjected to experimental AMI

The ratio 3-chlorotyrosine/Tyrosine was assessed by LC-ms. Internal standards, labelled tyrosine (13C9, 15N, L-Tyr) and 3-chlorotyrosine (13C9, 15N, L-ClTyr) were added to each sample for ratio calculations. Panels A-D show ion chromatograms for a SHAM sample where typically: L-Tyr eluted at ~2.9 min (Panel A, top image) with multiple reaction monitoring (MRM) transitions of mass to charge (m/z): 192 -> 98; 192->145; 192-> 174 (Panel A, bottom image). L-Cl-Tyr eluted at ~4.0 min (Panel B, top image) with MRM transitions of m/z: 226->179; 226->144; 226->208 (B, bottom image). Representative tyrosine ion chromatograms for a CON and a 4-MetT sample are shown in panels E and G, respectively and corresponding 3-Cl-Tyr chromatograms in panels F and H, respectively. Tyrosine eluted at different acquisition times for different samples however, the MRM remained consistent m/z: 182 -> 91; 182->136; 182->165 (C, bottom image).Representative tyrosine ion chromatograms for a SHAM, CON and a 4-MetT sample are shown in panels C, E and G respectively and corresponding 3-Cl-Tyr chromatograms in panels D, F and H respectively. Similarly, 3-Cl-Tyr eluted at various acquisitions times, but the corresponding MRM transitions remained consistent m/z: 216->170; 216->135; 216->199 (D, bottom image). Abbreviations: L-Tyr, Labelled-tyrosine.

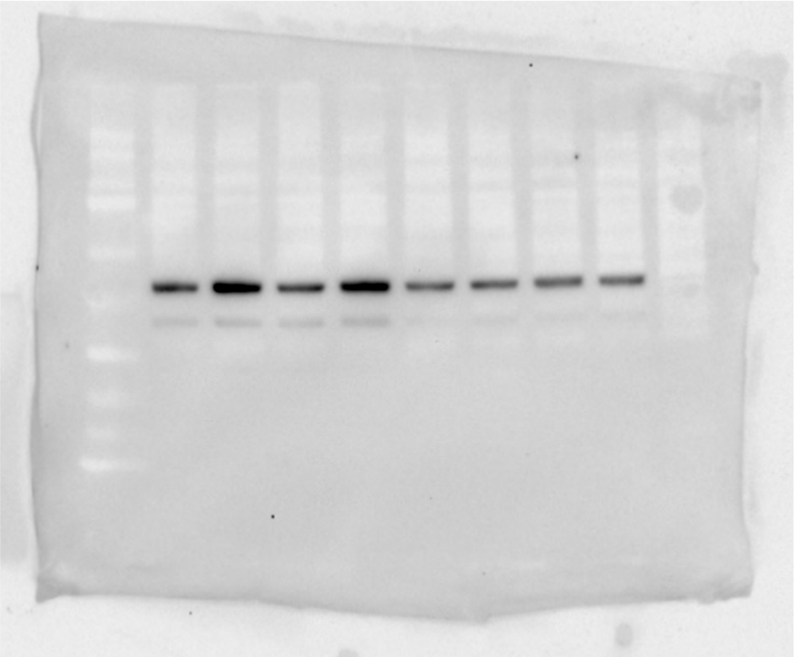

Supplementary Figure 2: Exemplar complete western blot for Figure 6 in the accompanying main manuscript.
For explanation of loading into lanes for this gel refer to figure 6 in the main manuscript.
